# Analysis of Molecular Epidemiological Characteristics of Porcine Reproductive and Respiratory Syndrome Virus Type 2 in Shandong Province from 2023 to 2025

**DOI:** 10.3390/vetsci13040314

**Published:** 2026-03-25

**Authors:** Zhenyang Li, Xinyuan Wang, Lin Jiang, Kexin Jin, Zhaoyang Feng, Jie Xu, Yesheng Shen, Fanliang Meng, Jianhua Qiu, Ning Li, Sidang Liu, Gang Wang

**Affiliations:** 1 Shandong Provincial Key Laboratory of Zoonoses, College of Veterinary Medicine, Shandong Agricultural University, Taian 271000, China; zhenyangli61@gmail.com (Z.L.);; 2 College of Veterinary Medicine, Nanjing Agricultural University, Nanjing 210000, China; 3 Veterinary Institute, Chinese Academy of Agricultural Sciences, Lanzhou Veterinary Research Institute, Lanzhou 730000, China

**Keywords:** PRRSV, Shandong province, molecular epidemiology, genetic diversity, recombination

## Abstract

Porcine Reproductive and Respiratory Syndrome Virus (PRRSV) poses a serious threat to the swine industry in China. As a major pig-producing province, Shandong requires continuous epidemiological monitoring of PRRSV. To elucidate the molecular epidemiology of the virus, 1621 clinical samples were collected from suspected cases across different regions of Shandong Province between 2023 and 2025, primarily from Tai’an, Linyi, Jining, and Liaocheng. RT-qPCR detection showed that the positive rate for PRRSV-2 was 20.05% (325/1621). Genetic analysis based on ORF5 and NSP2 genes indicated that Sublineage L1C (NADC30-like) was the dominant strain for 38.38% of ORF5 gene and 72.73% of NSP2 sequencing results. This was followed by Sublineage L8E and L1A and L5A strains. Key virulence-related mutations were identified at residues R13 and R151 in the GP5 protein, which are associated with enhanced pathogenicity. Additionally, variations in neutralizing epitope and the number of N-glycosylation sites (ranging from 2 to 5 per strain) suggested potential immune evasion. Notably, 26.79% (15/56) of sequenced samples showed discordant ORF5 and NSP2 genotyping results, indicating widespread recombination among PRRSV strains in Shandong Province. These finding demonstrated that the genetic diversity, high recombination frequency, and key amino acid variations in circulating PRRSV strains collectively undermine vaccine effectiveness. This study highlights the need to optimize vaccination strategies, enhance biosecurity measures, and implement effective disease control and elimination programs to reduce the impact of PRRSV in Shandong Province.

## Simple Summary

In this study, the researchers collected samples from sick pigs at many farms in Shandong Province between 2023 and 2025 and tested them to see if they carried the virus. Then, they examined important parts of the virus that control how harmful it is and how well it can escape the pig’s immune system and protection from vaccines. The results showed that several different lineages are circulating at the same time, that the virus often mixes its genetic material, and that key positions in its structure are changing in ways that may make it more harmful and harder to control with current vaccines. The findings will help improve vaccination plans, guide better farm hygiene and movement control, and support more accurate early warning systems.

## 1. Introduction

Porcine Reproductive and Respiratory Syndrome (PRRS), caused by the PRRSV virus (PRRSV), remains a significant infectious disease that continuously threatens the global swine industry [1]. In China, following their introduction into China in 2013 and 2017, respectively, PRRSV-2 NADC30 and NADC34 have undergone extensive recombination with endemic HP-PRRSV-like strains, leading to the persistent circulation of recombinant viruses in pig farms. Genetic variation and recombination among these strains have resulted in reduced vaccine efficacy, complicating epidemic prevention and control efforts [2,3]. As a major pig-producing region in China, Shandong Province plays a critical role in the national swine industry. In particular, the recent common practice of purchasing and fattening weaned piglets from various regions across the country highlights the importance of understanding the prevalence and molecular characteristics of PRRSV in Shandong. Such knowledge is essential for formulating effective national strategies for PRRS control and prevention.

In recent years, the evolutionary dynamics of PRRSV-2 have been notable for a continual increase in genetic diversity and mutation rate, contributing to a complex epidemiological landscape. In Shandong Province, PRRSV exhibits distinctive regional patterns, with PRRSV-2 maintaining dominance, particularly the NADC30-like and NADC34-like subtypes [4]. Owing to their pathogenicity, capacity for immune evasion, and propensity for genetic recombination, these strains cause persistently high morbidity and mortality rates in swine herds [4,5,6]. The concurrent risk of viral recombination and co-infection of other pathogens complicate disease prevention and control. Molecular variation and recombination in PRRSV not only compromise the efficacy of existing vaccines but are also closely associated with the clinical challenges such as immunosuppression and frequent secondary infections in swine herds [7,8,9].

This study systematically investigated the molecular epidemiological characteristics of PRRSV using positive clinical samples collected from pig farms in Shandong Province between 2023 and 2025. Through phylogenetic evolution and amino acid variation analysis, we elucidated the spatiotemporal distribution of viral strains, mutations in key gene loci, and their correlations with pathogenicity. The aim of this study was to provide a scientific basis for accurately assessing the risk of PRRS prevalence in Shandong Province, optimizing vaccine strategies, and enhancing biosecurity protocols. This is significant for improving the efficiency of regional PRRS control and also offers a theoretical foundation for effective epidemic management nationwide.

## 2. Materials and Methods

### 2.1. Source of the Animals

These samples were collected from various commercial pig farms in different cities in Shandong Province, China.

### 2.2. Sample Collection and Processing

Between 2023 and 2025, a total of 1621 suspected PRRSV infection samples were collected from different cities in Shandong Province (Table 1). The samples consisted of blood from live diseased pigs, as well as nasal swabs, lung tissues and lymph nodes obtained from deceased pigs. All samples were homogenized in sterile PBS and prepared as a suspension for subsequent detection. RNA was extracted using an automated nucleic acid extraction instrument (NPA-32P, Hangzhou Bior Technology Co., Ltd. is based in Hangzhou, Zhejiang, China) in accordance with the instructions of the RNA extraction and purification kit (Hangzhou Bior Technology Co., Ltd.) and further identified by a real-time fluorescent RT-PCR kit for detecting swine reproductive and respiratory syndrome virus (American strain) (Qingdao Jiazhi Biotechnology Co., Ltd. is based in Qingdao, Shandong, China).

### 2.3. Sequencing

Based on the conserved region sequences of ORF5 and NSP2 of PRRSV-2 from NCBI GenBank “https://www.ncbi.nlm.nih.gov/ (accessed on 25 March 2023)”, two primer pairs were designed for sequencing (Table 2). RT-PCR was performed in a 25 µL reaction system containing 12.5 µL of 2× One Step Mix (Dye Plus), 1 µL of One StepEnzyme Mix, 1 µL each of forward and reverse primers, 2 µL of RNA template, and 7.5 µL of RNase-free ddH_2_O. The reaction conditions are as follows: reverse transcription at 50 °C for 30 min; pre-denaturation at 95 °C for 3 min; 32 cycles of denaturation at 95 °C for 30 s; annealing at 58 °C for 30 s; and extension at 72 °C for 1 min, followed by a final extension at 72 °C for 7 min. PCR products were analyzed by 1% agarose gel electrophoresis and sent to Tsingke Biotechnology Co., Ltd. is based in Beijing, China for sequencing.

### 2.4. Gene Sequence Analysis

PRRSV-2 is divided into 11 main lineages (L1 to L11). These lineages include 21 Sublineages for finer resolution, such as L1A–L1F, L1H–L1J (9 in L1), L5A–L5B (2 in L5), L8A–L8E (5 in L8), and L9A–L9E (5 in L9).

Positive RT-PCR samples were subjected to sequencing of the ORF5 gene and a partial segment of the NSP2 gene. Reference sequences of PRRSV Sublineage L1A, L1C, L5A, and L8E, as well as lineage 3, were obtained from the NCBI database. Phylogenetic trees of PRRSV were constructed based on either ORF5 or NSP2 sequences. Multiple sequence alignment was performed using the FFT-NS-1 algorithm in MAFFT v7.526. A phylogenetic tree was subsequently built with the TVM + F + R4 model in IQTREE v2.4.0. Finally, the tree was visualized and refined using ITOL v7 “https://itol.embl.de/ (accessed on 15 October 2025)” [10,11].

### 2.5. Analysis of Amino Acid Mutations and Glycosylation Sites of ORF5

Sequence analysis was performed to identify mutations and glycosylation sites. Specifically, sequence alignments against the reference strain were conducted using MegAlign to pinpoint mutations, while N-glycosylation sites were predicted using NetNGlyc 1.0 “https://services.healthtech.dtu.dk/services/NetNGlyc-1.0/ (accessed on 25 October 2025)”.

### 2.6. Analysis of NSP2 Gene Deletion

Sequence alignment between the obtained sequences and the reference strain was performed using MEGA 7, followed by an analysis to identify missing sites.

## 3. Results

### 3.1. Genetic and Evolutionary Analysis of the ORF5 Gene and the NSP2 Gene

**Figure 1:**
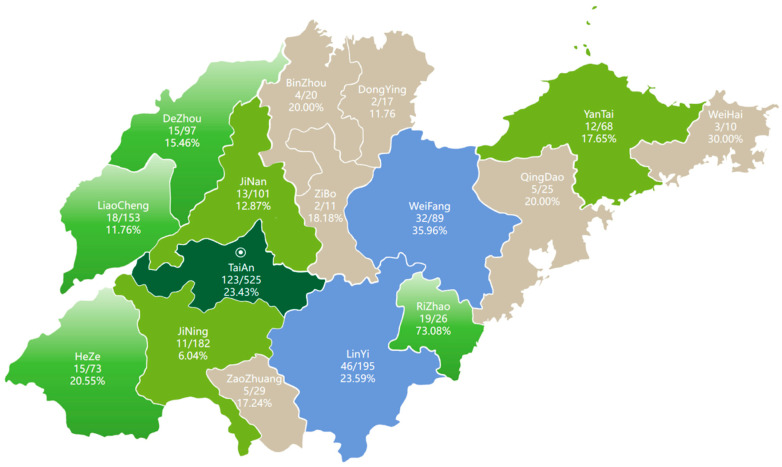
Map of sample collection and distribution in Shandong Province from 2023 to 2025 (the number represents the positive sample /number of samples collected and positive rate).

**Figure 2:**
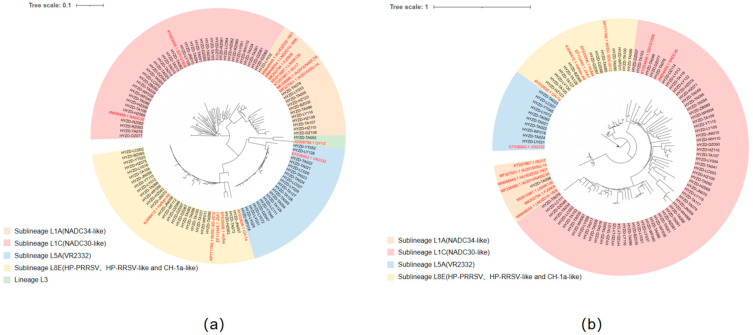
(**a**) Phylogenetic tree based on PRRSV ORF5 sequence. (**b**) Phylogenetic tree based on PRRSV NSP2 sequence. Phylogenetic tree based on ORF5 and NSP2 Genes. Red text indicates reference strains.

PRRSV was detected by RT-qPCR in all sampled regions, with an overall positivity rate of 20.05% (325/1621; 95% CI: 18.06–22.14%). Regional positivity rates showed substantial heterogeneity, ranging from 6.04% in Jining (11/182; 95% CI: 3.06–10.56%) to 73.08% in Rizhao (19/26; 95% CI: 52.21–88.43%) (Figure 1). Because of the heterogeneous sample sizes across regions and the relatively small numbers in some areas, exact 95% confidence intervals were calculated to provide a more robust estimate of regional positivity. Sequencing analysis was performed on samples that tested positive by RT-PCR. Using specific primers targeting the PRRSV ORF5 gene, a total of 99 sequences were obtained. Among these, 38 sequences belonged to Sublineage L1C, 12 to Sublineage L1A, 21 to Sublineage L5A, 27 to Sublineage L8E, and 1 to lineage 3. The proportions were 38.38% (38/99), 12.12% (12/99), 21.21% (21/99), 27.27% (27/99), and 1.01% (1/99), respectively (Figure 2a).

A total of 88 sequences were obtained by sequencing the PRRSV NSP2 gene. Among these, 64 sequences were classified as Sublineage L1C, 1 as Sublineage L1A, 10 as Sublineage L5A, 13 as Sublineage L8E. The proportions were 72.73% (64/88), 1.14% (1/88), 11.36% (10/88), and 14.77% (13/88), respectively. No lineage 3 was detected (Figure 2b).

### 3.2. Analysis of Nucleotide and Deduced Amino Acid Homology of ORF5

The PRRSV ORF5 gene sequence was analyzed using Megalign software and compared with the nucleotide and deduced amino acid sequences of six reference strains (CH-1a, VR2332, JXA1, NADC30, NADC34, and QYYZ). The sequenced PRRSV ORF5 nucleotide sequences exhibited high homology with PRRSV-2 JXA1, a highly pathogenic PRRSV-2 strain that emerged in China in 2006, with nucleotide identity at the genomic level ranging from 82.4% to 99.5%. Similarly, the deduced amino acids sequences showed high homologous to JXA1, ranging from 67.3% to 99.0% (Table 3).

### 3.3. Analysis of Amino Acid Site Mutations Deduced from the ORF5 Gene

**Figure 3:**
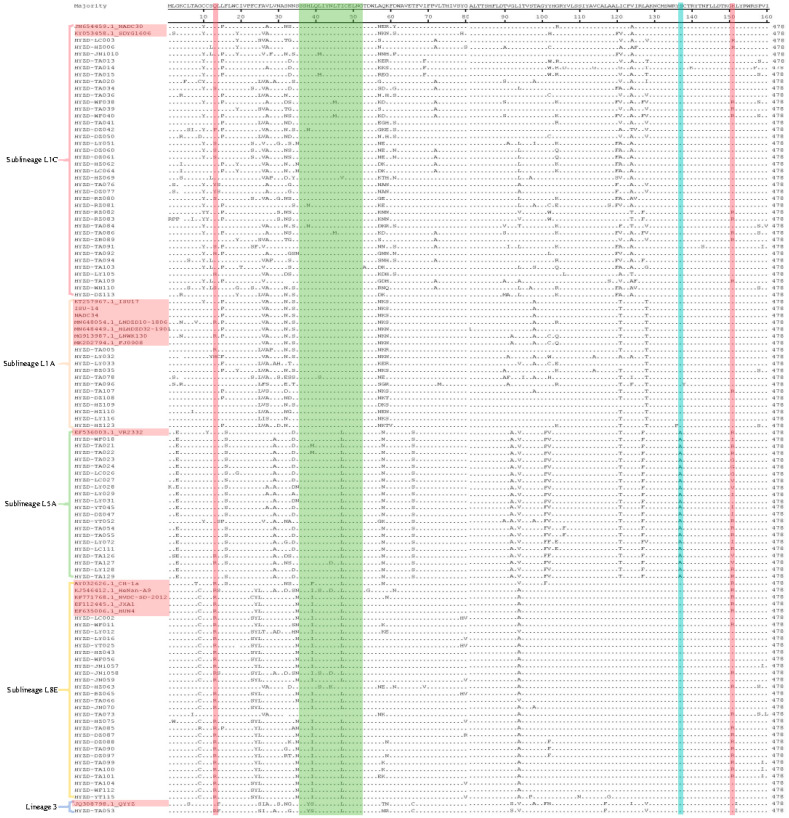
Analysis based on the major amino acid mutation sites of the PRRSV ORF5 gene. Red indicates the 13th and 151st amino acid, green indicates the amino acids 36–52, and blue indicates the 137th amino acid.

The MegAlign module of the DNASTAR lasergene software was used to analyze the deduced amino acid mutation sites of the 99 ORF5 genes in this study, along with those of some reference strains. The 13th and 151st amino acids encoded by ORF5 are associated with viral virulence. Among the strains, 29 have an amino acid of R at the 13th position: one belongs to Sublineage L1A, two belong to Sublineage L5A, one belongs to Lineage 3, and the remaining 25 belong to Sublineage L8E. In addition, 31 strains have an amino acid of R at the 151st position: 10 belong to Sublineage L1C, one to Sublineage L1A, eight to Sublineage L5A, and the remaining 12 to Sublineage L8E. A total of 10 strains have R at both positions; all belong to Sublineage L8E (Table 4; Figure 3).

The amino acids 36–52 encoded by ORF5 constitute the neutralization epitope region, a key region on the viral surface. Antibodies neutralize the virus by recognizing and binding to this region. This study found that 34 strains have mutations in the neutralization epitope region: nine belong to Sublineage L1C, one to Sublineage L1A, ten to Sublineage L5A, 26 to Sublineage L8E, and one to Lineage 3. The amino acids H38, I42, Y43, and N44 are identified as major antigenic recognition sites of the GP5 protein. Among these, three of Sublineage L1C have H38→N38, and one of Sublineage L1A and 2 of Sublineage L8E have N44→D/K44. Meanwhile, L39, Q40, and L41 amino acids are identified as antibody binding sites: 25 of Sublineage L8E have L39→I39, and one of Lineage 3 has L39→S39; two of Sublineage L1C have L41→M41, and one of Sublineage L1A and two of Sublineage L8E have L41→S41. Additionally, three of Sublineage L1C have L45→M45, and 21 of Sublineage L5A, 25 of Sublineage L8E, and one of lineage 3 has I47→L47 (Table 4; Figure 3).

The 137th amino acid (A137) of GP5 is a unique site of the vaccine strain and is considered a key marker for distinguishing wild-type strains from vaccine strains. In this study, 10 strains all carried the S137→A137 mutation and belong to Sublineage L5A (Table 4; Figure 3).

### 3.4. Analysis of N-Glycosylation Sites of the ORF5 Gene

**Figure 4:**
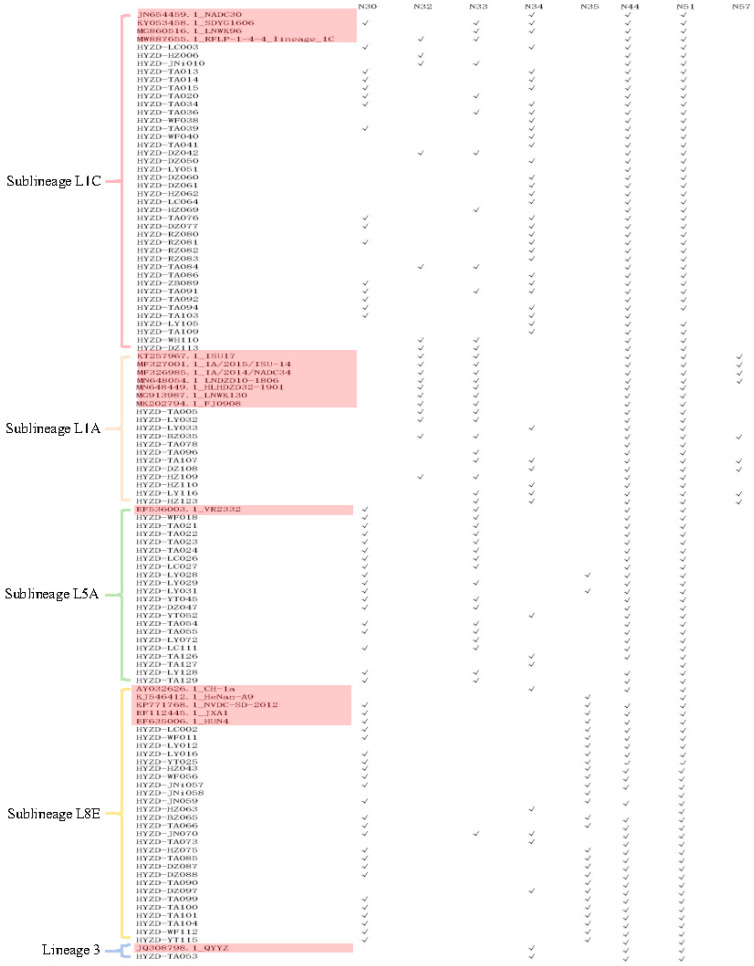
Comparation of potential N-glycosylation sites (NGSs) on GP5 protein.Red indicates reference strains.

ORF5 contained multiple N-glycosylation sites across all Sublineages. The majority of strains (59/112, 52.7%) had four NGSs, followed by 42/112 (37.5%) with three sites. Sublineage L1C exhibited the greatest diversity in NGS number, ranging from 2 to 5 sites (Table 5; Figure 4).

### 3.5. Analysis of Amino Acid Site Deletions in the NSP2 Gene

**Figure 5:**
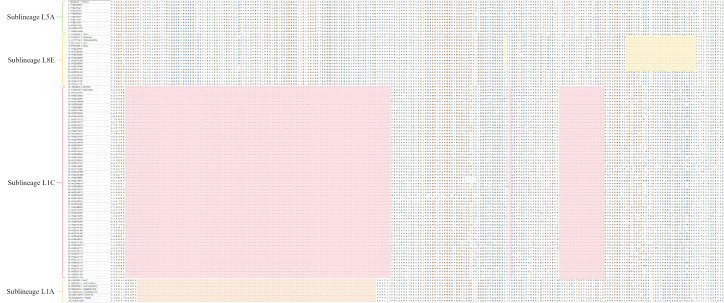
Analysis based on the major amino acid deletion sites of the PRRSV NSP2 gene. Red represents the L1C deletion site, yellow represents the L8E deletion site, and orange represents the L1A deletion site.

Using MAGE 7.0.26 software, we analyzed amino acid deletions in the partial NSP2 gene (900 aa at positions 2252-3152) sequence of 88 strains investigated in this study and the corresponding amino acid sites from selected reference strains of NCBI. Several characteristic deletions were identified. Among the 64 strains belonging to Sublineage L1C, the Nsp2 region exhibited molecular features consistent with the NADC30 strain, characterized by a discontinuous deletion of 131 aa at positions 323–433 (111aa), 484 (1aa), and 505–523 (19aa). In one strain of Sublineage L1A, the Nsp2 region display a deletion pattern identical to that of NADC34 strain, featuring a continuous 100 aa deletion at positions 328–427. For the 13 strains of Sublineage L8E, the Nsp2 region matched the molecular profile of the JXA1 strain, with a discontinuous deletion of 30 aa at positions 481 (1aa) and 532–560 (29aa). In contrast, no aa deletions were detected in the NSP2 region of 10 strains from Sublineage L5A (Figure 5).

### 3.6. Evidence of Lineage Recombination Between NSP2 and ORF5 Genes Identified in Multiple Viral Strains

A total of 56 samples were successfully sequenced for both the NSP2 and ORF5 genes. Among these, 15 samples (26.79%) exhibited discordant lineage classification results (Table 3). The distribution of these discrepancies was as follows: in eight strains (53.33%), ORF5 was classified as Sublineage L1A while NSP2 belonged to L1C; in five strains (33.33%), ORF5 was assigned to L8E and NSP2 to L1C; one strain (6.67%) showed ORF5 from L1A and NSP2 from L8E; and another single strain (6.67%) had ORF5 from L5A and NSP2 from L1C (Table 6).

## 4. Discussion

Porcine Reproductive and Respiratory Syndrome Virus (PRRSV) remains one of the most economically significant pathogens impacting the global swine industry. In this study, a total of 1621 samples from PRRS-suspected pigs were collected from various pig farms in Shandong Province from 2023 to 2025 and yielded 325 PRRSV-positive specimens, with a positivity rate of 20.05%, confirming that PRRSV is still a significant pathogen in the region. Phylogenetic analysis of successfully sequenced ORF5 (n = 99) and NSP2 (n = 88) genes revealed that NADC30-like strains (Sublineage L1C) remained dominant, accounting for 38.38% and 72.73% of the ORF5 and NSP2 sequences, respectively. Meanwhile, Sublineages L5A and L8E showed emerging trends, comprising 21.21% and 27.27% of ORF5 and 11.36% and 14.77% of NSP2 sequences. Consistent with prior findings, these results demonstrate that NADC30-like strains continue to prevail, and the emergence of Sublineages L5A and L8E suggests a complex and evolving PRRSV epidemiology in Shandong in the coming years [4,5,12].

The ORF5 gene encodes the major envelope glycoprotein GP5. Homology analyses demonstrated that the sequenced ORF5 nucleotides and deduced amino acids shared 82.4–99.5% and 67.3–99.0% identity, respectively, with the highly pathogenic PRRSV JXA1 strain. This suggests that the continued circulation and evolution of JXA1-like strains retain core virulence determinants. Amino acid substitutions at key positions within GP5 are known to modulate viral pathogenicity and antigenicity [4,13,14,15]. In this study, 29 strains exhibited an arginine (R) at position 13, and 31 at position 151—both residues associated with enhanced virulence. Notably, 12 strains harbored R at both sites, predominantly within Sublineage L8E, indicating that this lineage may possess elevated pathogenic potential [16,17]. Our analysis is limited to genetic data, and therefore predictions regarding virulence remain speculative. Further experimental studies, including neutralization epitope and challenge tests, are necessary to confirm these findings. Moreover, mutations within the neutralizing epitope (aa 36–52), were identified in 34 strains across all Sublineages. Critical antigenic recognition sites—H38, I42, Y43, and N44—displayed non-conservative substitutions that are likely to impair antibody binding [16,18,19]. The detection of the A137 mutation (a hallmark of vaccine strains [20]) in ten L5A strains indicates either the circulation of vaccine-derived viruses or reversion events. This observation underscores the risk associated with modified live virus (MLV) vaccines, including the potential for recombination with field viruses or reversion to virulence, thereby complicating control and eradication efforts [21,22].

N-glycosylation sites (NGSs) within GP5 contribute to immune evasion by masking antigenic epitopes from antibody recognition [23,24]. The variable number of NGSs across Sublineages, ranging from two to five, suggests ongoing adaptation of the viral glycan shield. Strains with fewer glycosylation sites may exhibit altered immunogenicity profiles, potentially influencing both vaccine efficacy and viral fitness under immune selection [14,25,26,27,28,29].

The NSP2 gene, recognized for its hypervariability and role in viral polyprotein processing and host innate immune modulation [30], displayed considerable genetic heterogeneity. Characteristic deletion patterns within NSP2 aligned with those of reference strains: L1C strains exhibited NADC30-like discontinuous deletions, L1A matched the NADC34 deletion profile, and L8E strains conformed to the JXA1 pattern. In contrast, L5A strains lacked deletions, resembling classical PRRSV strains. In this study, the inter-lineage recombination between NSP2 and ORF5 was also observed. Among the 56 strains successfully sequenced for both genes, 15 (26.79%) displayed discordant lineage classifications. The most prevalent recombination pattern involved ORF5-L1A paired with NSP2-L1C (53.33%), followed by ORF5-L8E with NSP2-L1C (33.33%). Although indications of PRRSV recombination were observed, the current data allow only partial detection of these events. These results confirm that recombination between divergent PRRSV strains is occurring in the field, contributing to genomic plasticity and potentially generating novel variants with altered phenotypic properties [31]. Similar inter-lineage recombination patterns between NSP2 and ORF5 have been reported in earlier research on PRRSV-2 strains, particularly involving lineages 1 and 8, which aligns with our findings of NSP2-L1C dominance as a recurrent parental source. For instance, a 2022 analysis of 949 global sequences from 1991 to 2021 identified frequent NSP2 and ORF5 exchanges, with lineage 1 emerging as the primary recombinant backbone post-2012, mirroring the L1C prevalence here [32].

## 5. Conclusions

In conclusion, the PRRSV epidemic in Shandong is predominantly characterized by PRRSV-2 strains, with Sublineages L1C and L1A as the primary subtypes. The increasing prevalence of L8E and L5A, along with frequent mutations in key virulence and neutralizing epitopes (e.g., ORF5 R13, R151, and GP5 N-glycosylation sites), may relate to viral pathogenicity and likely contributes to immune escape. Moreover, widespread genetic recombination among circulating strains and mixed infections underscores the necessity of optimizing vaccination strategies and strengthening biosecurity and regional surveillance for effective control.

## Figures and Tables

**Table 1:** Suspected PRRSV infection sample background information.

Area	Number of Pathogen Collection Samples	Number of Pig Farms Sampled for Pathogens	Vaccine Immunization Rate (%)
Jinan	101	19	88.12
Qingdao	25	7	76.00
Zibo	11	2	90.91
Zaozhuang	29	8	82.76
Dongying	17	4	88.24
Yantai	68	12	88.24
Weifang	89	15	88.76
Jining	182	27	87.91
Taian	525	56	82.86
Weihai	10	2	100.00
Rizhao	26	5	88.46
Binzhou	20	5	90.00
Dezhou	97	17	74.23
Liaocheng	153	26	88.89
Linyi	195	30	78.98
Heze	73	9	71.23

**Table 2:** List of primers used in this study.

Names	Primer Sequence (5′-3′)	Length (bp)
PRRSV-ORF5-F	ACCTGAGACCATGAGGTGGG	971
PRRSV-ORF5-R	GCAAGCACAAACGGCATCTG	
PRRSV-NSP2-F	ATGTTGTGCTTCCTGGGGTTG	1046
PRRSV-NSP2-R	CTTGACAGGGAGCTGCTTGA	

**Table 3:** List of homology alignments between the PRRSV-positive field virus and some reference virus ORF5 nucleotides and encoded amino acids.

Reference Virus Name	Area	Year	Accession Number	Isolation of Viral nt Homology	Isolation of Viral aa Homology
CH-1a	China	1996	AY032626.1	82.4–95.4%	70.3–93.1%
VR2332	USA	1992	EF536003.1	76.9–99.5%	64.9–99.0%
JXA1	China	2006	EF112445.1	82.4–99.5%	67.3–99.0%
NADC30	USA	2008	JN654459.1	75.6–93.4%	63.9–95.0%
NADC34	USA	2014	MF326985.1	75.8–95.7%	64.4–95.5%
QYYZ	China	2011	JQ308798.1	73.6–85.2%	61.4–86.1%

**Table 4:** Key amino acid mutations in ORF5 associated with virulence, antigenicity, and vaccine marker.

Position	Sublineage L1A	Sublineage L1C	Sublineage L5A	Sublineage L8E	Lineage 3	Total
13	1	0	2	25	1	29
151	1	10	8	12	0	31
36–52	1	9	10	26	1	47
137	0	0	10	0	0	10

**Table 5:** Distribution of N-glycosylation sites (NGSs) in ORF5 across PRRSV.

Sublineage	2 Sites	3 Sites	4 Sites	5 Sites	Total Strains
L1A	1	18	4	3	26
L1C	1	18	17	2	38
L5A	1	3	17	0	21
L8E	2	3	21	1	27
Total	5	42	59	6	112

**Table 6:** List of the comparative analysis of two gene fragments ORF-5 and NSP2 of the PRRSV-positive field samples.

Number of Samples	According to ORF5	According to NSP2
HYZD-LC002	Sublineage L8E	Sublineage L1C
HYZD-TA005	Sublineage L1A	Sublineage L1C
HYZD-LY012	Sublineage L8E	Sublineage L1C
HYZD-JN059	Sublineage L8E	Sublineage L1C
HYZD-TA066	Sublineage L8E	Sublineage L1C
HYZD-JN070	Sublineage L1A	Sublineage L1C
HYZD-TA078	Sublineage L1A	Sublineage L1C
HYZD-TA096	Sublineage L1A	Sublineage L1C
HYZD-TA107	Sublineage L1A	Sublineage L1C
HYZD-HZ109	Sublineage L1A	Sublineage L1C
HYZD-HZ110	Sublineage L1A	Sublineage L1C
HYZD-LC111	Sublineage L5A	Sublineage L1C
HYZD-YT115	Sublineage L8E	Sublineage L1C
HYZD-LY116	Sublineage L1A	Sublineage L1C
HYZD-TA127	Sublineage L1A	Sublineage L8E

## Data Availability

The datasets generated and/or analyzed during the current study are available in the The National Center for Biotechnology Information repository, “https://www.ncbi.nlm.nih.gov/ (accessed on 26 November 2025)”.
